# Branch retinal vein occlusion associated with platelet activation

**DOI:** 10.3906/sag-1807-223

**Published:** 2019-02-11

**Authors:** Emrullah BEYAZYILDIZ, Mehmet ÇITIRIK, Mert ŞIMŞEK, Özlem BEYAZYILDIZ, İbrahim C. HAZNEDAROĞLU

**Affiliations:** 1 Samsun Training and Research Hospital, University of Health Sciences, Samsun Turkey; 2 Ankara Ulucanlar Eye Education and Research Hospital, University of Health Sciences, Ankara Turkey; 3 Department of Hematology, Faculty of Medicine, Hacettepe University, Ankara Turkey

**Keywords:** Branch retinal vein occlusion, mean platelet volume, plateletcrit, platelet distribution width, platelet parameters, retina

## Abstract

**Background/aim:**

The aim of this study is to study subclinical platelet activation by detecting three important platelet activation parameters of mean platelet volume (MPV), platelet distribution width (PDW), and plateletcrit (PCT) in patients with branch retinal vein occlusion (BRVO) in comparison to those in healthy control subjects.

**Materials and methods:**

This prospective study included 43 patients with BRVO (Group 1) and 40 control subjects (Group 2). The levels of MPV, PDW, and PCT were measured in both of the studied groups.

**Results:**

The mean serum level of MPV value was 7.64 ± 0.64 in Group 1 and 7.39 ± 0.42 in Group 2. Mean serum level of PDW was 15.01 ± 1.56 in Group 1 and 14.43 ± 1.03 in Group 2. Mean serum PCT value was 0.19 ± 0.05 in Group 1 and 0.16 ± 0.04 in Group 2. MPV, PDW, and PCT levels were significantly increased in BRVO patients (P < 0.05).

**Conclusion:**

Subclinical platelet activation reflected by MPV, PDW, and PCT may have an impact on the genesis of vessel occlusion in BRVO. The results may be important for the clinical management of patients with BRVO.

## 1. Introduction 

Retinal venous occlusion is the second most common retinal vascular disorder after diabetic retinopathy causing visual loss (1). Branch retinal vein occlusion (BRVO) is a frequent retinal vascular disease with a yearly incidence of 2.14/1000 in the population over 40 years of age (2). BRVO is predisposed via various systemic and local factors (2). Hypertension and end-organ damage caused by diabetes mellitus contribute to arteriosclerosis, atherosclerosis, and endothelial dysfunction, which seem to be major risk factors for BRVO (3). Ophthalmic risk factors are ocular hypertension, glaucoma, higher ocular perfusion pressure, and changes in the retinal arteries (4). Underlying mechanisms for etiopathogenesis of BRVO are degenerative changes of the vessel wall, compression of the vein by arteriole at the arteriovenous (AV) crossing, and disturbed hematological factors (5). Endothelial dysfunction and platelet activation lead to occlusion of branch retinal veins (6,7).

Platelets have a very important role in the regulation of hemostasis and vascular integrity. Platelet indices such mean platelet volume (MPV), platelet distribution width (PDW), and plateletcrit (PCT) are standard indicators of platelet function in disease pathophysiology. The alterations in platelet parameters may affect the prothrombotic status of a patient. The platelet markers have been associated with several pathological conditions, such as increased levels of MPV shown as a risk factor for myocardial infarction and stroke (8,9). In a recent study, MPV was also shown to be increased in BRVO patients (10). To the best of our knowledge, the platelet activation parameters including PDW and PCT have not been studied in BRVO patients previously. The aim of this study is to assess the possible interrelationships of BRVO with platelet indices MPV, PDW, and PCT in order to detect subclinical platelet activation.

## 2. Materials and methods

This study was carried out with 43 patients with BRVO between September 2013 and January 2015. All procedures adhered to the tenets of the Declaration of Helsinki, and local approval was received from the Ethical Committee of Ankara Dışkapı Training and Research Hospital. Informed consent was obtained from each patient after explanation of the research purposes. All patients were Turkish Caucasians. This study is registered with the Australian New Zealand Clinical Trials Registry, number ACTRN12616001246471.

The patients were included within two study groups based on the findings of clinical ocular examination. The first group included 43 BRVO patients (Group 1); the second group included 40 adult subjects serving as controls with no history of ocular and systemic disease except for hypertension and diabetes mellitus (Group 2). There were no personal or familial histories of thrombotic disease in the patients and control cases. The absence of thrombotic events or a family history of thrombosis was confirmed by means of a verified questionnaire. Patients and controls using any medication including corticosteroid and immunosuppressive therapy, who had smoking or drinking habits, or who had a history of systemic inflammatory and ocular disease were excluded. All of the patients and controls had normal liver and renal function tests and electrolytes. In order to standardize the hemostatic system parameter measurements, all sampling procedures were performed in the morning hours with fasting. The blood was collected in EDTA vacuum tubes with minimum stasis and examined within 60 min. 

A complete ophthalmological examination including best corrected visual acuity, intraocular pressure measurement with Goldmann applanation tonometry, biomicroscopic examination, and dilated pupil examination of the posterior segment was performed in both groups. Subclinical BRVO was not observed in the control group.

The statistical analysis was performed by t-test according to Bonferroni procedures for multiple comparisons. The significance level was determined as P < 0.05. Differences between two groups for age and sex were evaluated using Mann–Whitney U tests. All statistics in this study were analyzed using SPSS 18.0 for Windows (SPSS Inc., Chicago, IL, USA). 

## 3. Results

Eighty-three individuals were included in this clinical research. There were 49 women (59.1%) and 34 men (40.9%). The mean age was 59.6 ± 8.1 years (mean ± SD) in Group 1 and 61.3 ± 12.8 in Group 2. There were no difference in terms of age or sex (P = 0.5) (Table 1).

**Table 1:** Demographic data of the groups.

	Group 1 (n = 43)	Group 2 (n = 40)	P
Age (years)	61.37 ± 12.87	59.65 ± 8.15	0.47
Sex (male/female)	18/25	16/24	0.17

Of the 43 BRVO patients, 25 (58.1%) had hypertension and 12 (27.9%) had diabetes mellitus. The coexistence of hypertension and diabetes mellitus was present in 6 patients (13.9%). The location of vein occlusion was superotemporal in 28 eyes (65.1%), inferotemporal in 14 (32.5%), and inferonasal in 1 (2.3%). The mean best-corrected visual acuity was 20/80 (range: 20/2000 to 20/25) in the BRVO group. Biochemical parameters including glucose, lipid, and homocysteine values and clotting, plasma viscosity, and inflammatory markers were normal.

Of the 40 control subjects, 20 (52.5%) had hypertension, 9 (22.5%) had diabetes, and 4 (10%) had both hypertension and diabetes mellitus.

MPV levels showed a marked elevation in our BRVO patients (P = 0.03) compared with controls. The mean serum level of MPV was 7.64 ± 0.64 in Group 1 and 7.39 ± 0.42 in Group 2 (Table 2).

**Table 2:** The mean values of mean platelet volume, plateletcrit, and platelet distribution width and comparison of platelet parameters (mean ± standard deviation) for groups.

	Group 1 (n = 43)	Group 2 (n = 40)	P
MPV (fL)	7.64 ± 0.64	7.39 ± 0.42	0.03
PDW (%)	15.01 ± 1.56	14.43 ± 1.03	0.01
PCT (%)	0.19 ± 0.05	0.16 ± 0.04	0.04

MPV: Mean platelet volume, PDW: platelet distribution width, PCT: plateletcrit.

Increments in the serum level of PDW were observed in BRVO cases (P = 0.01) when compared to the control group. The mean serum level of PDW was 15.01 ± 1.56 in Group 1 and 14.43 ± 1.03 in Group 2 (Table 2).

The mean PCT levels were 0.19 ± 0.05 in BRVO cases, and these were significantly higher than in controls (0.16 ± 0.04; P = 0.04) (Table 2).

## 4. Discussion

Branch retinal vein occlusion is a common cause of retinal vascular disorder and is said to be the second most common disease after diabetic retinopathy (11). Major pathogenetic factors confined to the pathogenesis of BRVO are abnormal hematological parameters, degenerative changes of the retinal vessel wall, and compression of the retinal vein at the AV crossing (12). Although multiple hematological parameters were discussed in the pathogenesis of BRVO, the role of coagulation parameters remains unclear. Thrombosis may develop due to platelet abnormalities, endothelial injury, increased procoagulant activity, and abnormal fibrinolysis. Larger platelets are more active and contain more intense granules and they have more powerful prothrombotic activity (13).

Increased MPV may have a role in the vessel occlusion process of BRVO. Platelet activation also correlates with increased MPV levels. The high value of MPV is an indication of increased thrombocyte size. Large platelets as diameters are more active, functional, and dense than small ones. Thus, higher MPV levels may increase the possibility of vascular complications (14,15). Previous studies showed that MPV was associated with situations such as coronary and peripheral artery disease, myocardial infarction, and cerebral ischemia (16–19).

PDW is also related to platelet activation and it is a more specific marker than MPV, because blood values of PDW are not high during simple platelet swelling (20). PDW shows variations in platelet size that may be indicators of active platelet release (21). PDW levels could be altered in several conditions and increased levels were observed in sickle cell patients with vaso-occlusive crisis (22,23). PDW may be a more specific marker than MPV for showing platelet activation and increased levels of PDW might show impaired deformability of thrombocytes and be related to microvascular resistance (24–26). Vagdatli et al. (20) found that PDW is a specific and simple marker for coagulation activation. Thus, increased PDW levels could be a risk factor and play a role in the pathogenesis of BRVO. Amin et al. (23) showed elevated levels of PDW during vaso-occlusive complications in sickle cell anemia patients. 

PCT is a marker of blood-circulating platelets in a unit volume. PCT reveals quantitative abnormalities of platelets and is calculated as platelets × MPV/107 (27). Akpinar et al. (13) found that PCT had a significant predictive value for saphenous vein graft disease and emphasized that it could be used as a marker for antiplatelet therapy to prevent graft atherosclerosis in patients undergoing bypass surgery. Onder et al. (10) showed decreased levels of platelets in hypertensive BRVO patients, but not significantly. In another previous study, PCT was correlated with C-reactive protein in chronic inflammatory diseases (28). An increased MPV level in patients with hypertension and diabetes mellitus without BRVO was reported previously (29). Therefore, our control subjects were selected from among individuals with a history of hypertension and diabetes mellitus.

In the present study, BRVO patients showed significantly higher levels of MPV, PDW, and PCT. The increased levels of these parameters may play a role in the pathogenesis of BRVO. Increment in these platelet parameters may increase activation and aggregation of platelets and increase vasoactive mediators’ secretion by these platelets such as thromboxane A2, resulting in vasoconstriction, endothelial dysfunction, and impaired blood flow, which results in microvascular occlusion. Some studies suggested that thrombophilic parameters are altered in groups of patients with BRVO and control groups (10,30). However, to the best of our knowledge, PDW and PCT have not been evaluated before in patients with BRVO.

MPV is calculated by dividing the PCT by the number of platelets, which is the same calculation as for the mean red cell volume, namely dividing hematocrit by the red blood cells, and therefore PCT is analogous to the red cell hematocrit (31). Red cell indices are widely used in clinics. On the other hand, platelet indices (other than the platelet count itself) are probably the most regularly ignored part of the automated complete blood count parameters (32). Positive studies and metaanalyses of automated platelet analyses focusing on coronary heart diseases highlighted long-term efforts about the validation of those ‘routine’ lab parameters in eye vessel occlusions including BRVO (33).

The pathogenesis of BRVO includes local microenvironmental factors, including degenerative changes of vascular endothelium, compression at the AV crossing, and others. Systemic hypercoagulable factors of disturbed hematological factors including platelet activity may also have impact as a triggering factor. The treatment of this challenging occlusive event may not rely solely on antiplatelet drugs. However, we must be aware of the ongoing subclinical thrombocyte activation during the clinical management of the patients with BRVO.

Squizzato et al. (34) investigated the best available evidence on the acute treatment and the secondary prevention of RVO with antithrombotic and fibrinolytic drugs. A search of the MEDLINE and EMBASE electronic databases up to January 2009 was performed. They suggested that antithrombotic therapy, in particular low-molecular-weight heparin, may be a part of the therapeutic armamentarium for patients with recent-onset RVO. Houtsmuller et al. (35) established a double-blind study to evaluate the platelet aggregation inhibiting effect of ticlopidine on the course of ocular vein occlusions. They determined that the effect of ticlopidine was most pronounced in patients with increased platelet aggregation and least obvious in cases of hyperlipidemia. Hypertension and diabetes did not apparently influence ticlopidine’s effects. The platelet aggregation inhibitor ticlopidine was found effective in the treatment of recent ocular vein occlusions.

There are several limitations in the present study. The limited number of patients is the most important limiting factor. Secondly, this study is based on a simple baseline determination that may not reflect a patient’s long-term status.

Based on the results of our present study, BRVO patients have significantly higher serum levels of MPV, PDW, and PCT than control subjects. MPV, PDW, and PCT seem to play an important role in the genesis of BRVO. Serial measurements of PDW, MPV, and PCT may be helpful in the diagnosis and prevention of BRVO. The sudden peak of these markers indicates platelet activation. The results may be important for the clinical management of patients with BRVO in everyday clinical practice since those platelet activation parameters are routinely detected in complete blood count analyses. Further large-scale and comprehensive studies are needed to support these results.

Based on the current data, if an ophthalmologist does have a concern that platelet activation could be impaired in a patient with BRVO, he or she could share the case with a hematologist if there is any sign of systemic platelet activation syndromes that potentially affect any micro- and/or macrocirculatory vessel systems. Antithrombotic, antiplatelet, anticoagulant, and profibrinolytic management strategies should then be determined for the given patient, as well as the hemostatic follow-up.
